# A PARTICIPATORY STUDY TO CO-DESIGN SOCIAL NORM NUDGES FOR IMPROVING MENTAL HEALTH HELP-SEEKING IN OLDER PEOPLE

**DOI:** 10.1093/geroni/igae098.3335

**Published:** 2024-12-31

**Authors:** Tianyin Liu, Jia Wang

**Affiliations:** The Hong Kong Polytechnic University, Hong Kong, Hong Kong; The Hong Kong Polytechnic University, Hong Kong, Hong Kong

## Abstract

Mental health help-seeking is often delayed in older people, and different theories have hypothesized diverse mechanisms to improve help-seeking. The theory of planned behavior (TPB) and nudge theory (NT) converge on social norms as promising targets for intervention. Despite accumulating evidence of social norm nudges in promoting health behaviors, no intervention has utilized social norms to promote mental health help-seeking in older adults. This study aims to (a) co-design social norm-nudges with different stakeholders and (b) pilot their feasibility and effects in improving mental health help-seeking intention in older people. The participatory study recruited ten older people, two social workers, and two experienced researchers to co-design the social norm nudges. All participants received three-hour training on TPB and NT principles, followed by ten interactive co-design workshops over three months. A set of 14 social norm nudges was designed and piloted among 20 older people for feasibility and effects. The participants completed a self-administered baseline questionnaire, received 14 social norm nudges over smartphones in two weeks (one message per day), completed the post-intervention questionnaire and provided feedback to the nudges afterwards. The primary outcomes were changes in subjective norms and help-seeking intention assessed using a revised Chinese version of the TPB questionnaire (C-TPB); secondary outcomes included perceived behavioral control, help-seeking attitude, perceived barriers to help-seeking (C-TPB subscales), and mental health assessed by PHQ-9, GAD-7 and UCLA-3. Preliminary results suggested the feasibility of the social norms to be delivered digitally and the effects in increasing help-seeking intention and attitude.

